# Molecular Mechanisms of Colon Cancer Progression and Metastasis: Recent Insights and Advancements

**DOI:** 10.3390/ijms22010130

**Published:** 2020-12-24

**Authors:** Ahmed Malki, Rasha Abu ElRuz, Ishita Gupta, Asma Allouch, Semir Vranic, Ala-Eddin Al Moustafa

**Affiliations:** 1 Department of Biomedical Science, College of Health Sciences, QU Health, Qatar University, Doha 2713, Qatar; ra1903138@student.qu.edu.qa (R.A.E.); aa1602752@student.qu.edu.qa (A.A.); 2 College of Medicine, QU Health, Qatar University, Doha 2713, Qatar; ishugupta28@gmail.com (I.G.); svranic@qu.edu.qa (S.V.); 3 Biomedical and Pharmaceutical Research Unit, QU Health, Qatar University, Doha 2713, Qatar; 4 Biomedical Research Center, QU Health, Qatar University, Doha 2713, Qatar

**Keywords:** colorectal cancer, molecular pathways, chromosomal instability, microsatellite instability, therapeutics

## Abstract

Colorectal cancer (CRC), the third most common type of cancer, is the second leading cause of cancer-related mortality rates worldwide. Although modern research was able to shed light on the pathogenesis of CRC and provide enhanced screening strategies, the prevalence of CRC is still on the rise. Studies showed several cellular signaling pathways dysregulated in CRC, leading to the onset of malignant phenotypes. Therefore, analyzing signaling pathways involved in CRC metastasis is necessary to elucidate the underlying mechanism of CRC progression and pharmacotherapy. This review focused on target genes as well as various cellular signaling pathways including Wnt/β-catenin, p53, TGF-β/SMAD, NF-κB, Notch, VEGF, and JAKs/STAT3, which are associated with CRC progression and metastasis. Additionally, alternations in methylation patterns in relation with signaling pathways involved in regulating various cellular mechanisms such as cell cycle, transcription, apoptosis, and angiogenesis as well as invasion and metastasis were also reviewed. To date, understanding the genomic and epigenomic instability has identified candidate biomarkers that are validated for routine clinical use in CRC management. Nevertheless, better understanding of the onset and progression of CRC can aid in the development of early detection molecular markers and risk stratification methods to improve the clinical care of CRC patients.

## 1. Introduction

Colorectal cancers (CRCs) encompass two types of highly aggressive and common types of cancers, namely, colon and rectal. Globally, while colon cancer is the fourth most common malignancy, rectum cancer is the eight most common one [1]. Collectively, CRCs present the third most commonly diagnosed form of cancer worldwide, accounting for 11% of all diagnosed cancer cases [1]. Additionally, CRC is the second most lethal cancer worldwide [2]. In this context, the prevalence of CRC cases varies from one geographical location to another, as Hungary has the highest incidence of CRC among males and Norway has the highest incidence amongst females [2]. Nevertheless, CRC is frequently diagnosed in men from Japan, South Korea, the Middle East region, and Slovakia, with mortality being highest in Saudi Arabia, Oman, and UAE [2].

Two molecular pathological classifications for CRC are described [3,4]. The Cancer Genome Atlas (TCGA) project first classified CRC into two groups using integrated molecular analysis (array-based and sequencing technologies) [3]. The first group is comprised of approximately hypermutated tumors (~16%) with either microsatellite instability (MSI) due to defective mismatch repair (MMR) (∼13%) or ultra-mutated tumors with DNA Polymerase Epsilon or Delta 1 (POLE or POLD1) exonuclease domain (proofreading) mutations (EDM) (∼3%) [3]. The second group consisted of non-hypermutated tumors (~84%), microsatellite stable (MSS) cancers with a high frequency of DNA somatic copy number alterations (SCNAs) and dysregulated Wnt pathway with frequent mutations in genes including *Adenomatous polyposis coli* (*APC*)*, Kirsten ras* (*KRAS*)*, Phosphatidylinositol-4,5-Bisphosphate 3-Kinase Catalytic Subunit Alpha* (*PIK3CA*)*, Small mothers against decapentaplegic 4* (*SMAD4*), and *Tumor protein 53* (*TP53*) [3].

On the other hand, Guinney et al. (2016) [4] aggregated gene expression datasets using 18 different CRC and described the four consensus molecular subtypes (CMS) of CRC: CMS1 (MSI-immune), CMS2 (canonical), CMS3 (metabolic), and CMS4 (mesenchymal) (Table 1).

To determine the extent of CRC spread, according to the American Joint Committee on Cancer, CRC staging is done according to the tumor-nodes-metastasis (TNM) system [5].

CRC pathogenesis is highly complex and diverse and is induced by several risk factors including sporadic, familial, and inherited [6]. Sporadic cases comprise 70% of CRC cases and are comprised of environmental and dietary factors (cigarette smoking, excessive alcohol consumption, sedentary lifestyle, obesity, and diets high in fat and low in fiber) [6,7]. Familial CRC cases comprise 25% of the cases and affect individuals with family history of CRC [6]. Lastly, genetic or inherited cases account for only 5–10% of the cases and are categorized based on the presence or absence of colonic polyps [6]. While diseases with polyposis include familial adenomatous polyposis (FAP) (OMIM#: 608456), MUTYH-associated polyposis (MAP) (OMIM#: 175100), hamartomatous polyposis syndromes (Peutz-Jeghers, juvenile polyposis (OMIM#: 175200) [8] and Cowden syndrome (OMIM#: 158350), diseases without polyposis are known as hereditary nonpolyposis CRC (HNPCC; Lynch syndrome (OMIM#: 120435)) [6]. Other risk factors involve the presence of existing diseases such long-standing inflammatory bowel diseases (IBD), either Crohn or ulcerative colitis [9].

On the other hand, genetic and epigenetic alterations are involved in events that lead to the initiation of neoplastic transformation of healthy epithelium that progresses toward the malignant stages; in 1990, Fearon and Vogelstein defined the genetic paradigm of CRC formation [10]. In their multistep genetic model, formation of CRC occurs due to accumulation of several genetic and epigenetic alterations in key genes involved in silencing of TSGs and activation of oncogenes [10,11,12]. In this regard, two major pathways were defined for CRC formation. One pathway involves inhibiting tumor suppressor genes’ (TSGs) expression and adenomatous polyposis coli (APC). This comprises 85% of all CRC and is mutated in the germline of patients with FAP. The other pathway is via mutational inactivation of proteins involved in MMR (MSH2, MLH1, and PMS2) and accounts for around 15% of all sporadic cases and HNPCC syndrome [10,11,12]. The different pathways are characterized by distinctive models of genetic instability, subsequent clinical manifestations, and pathological characteristics [11,12]. However, the majority of CRC cases follow the chromosomal instability (CIN) pathway, characterized by an extensive loss of heterozygosis (LOH) and gross chromosomal abnormalities [13,14].

Today, CRC is one of the leading causes of morbidity and represents a formidable health burden. Therefore, understanding the molecular and genetic features of its onset and progression is essential. This review focused on several novel cellular signaling pathways associated with CRC metastasis, including Wnt/β-catenin, p53, cyclooxygenase (COX), TGF-β/SMAD, nuclear factor kappa beta (NF-κB), Notch, vascular epidermal growth factor (VEGF), and Janus kinase/signal transducer and activator of transcription 3 (JAKs/STAT3) pathways. Additionally, targeted therapeutic agents developed based on these pathways are also briefly discussed.

## 2. Molecular Basis of Colorectal Cancer

**Figure 1:**
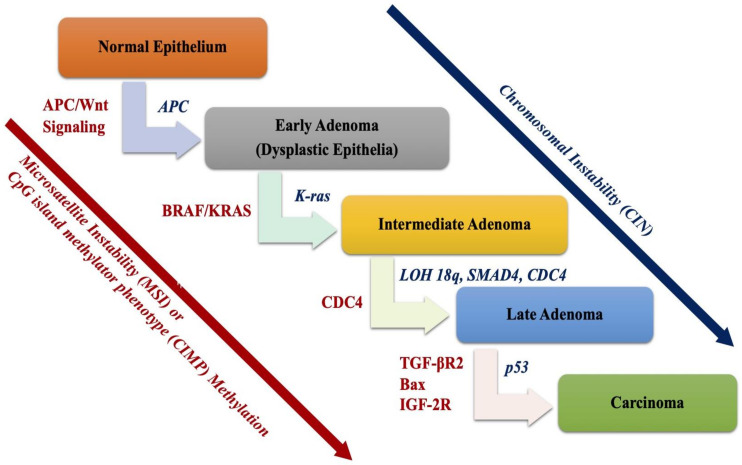
Multistep genetic model for colorectal adenocarcinoma sequence. There are three pathways regulating the adenocarcinoma sequence: chromosomal instability (CIN), microsatellite instability (MSI), and CpG island methylator phenotype (CIMP) hypermethylation.

As mentioned above, both genetic and epigenetic alterations are involved in regulating tumorigenesis of CRC [10]. The adenocarcinoma sequence was described much earlier, in 1980, when the transformation of normal colorectal epithelium to an adenoma and, finally, to an invasive and metastatic tumor was elucidated [10]. There are three major pathways involved in the genetic instability of CRC and its pathogenesis: chromosomal instability (CIN), microsatellite instability (MSI), and CpG island methylator phenotype (CIMP) pathways (Figure 1) [15].

### 2.1. Chromosomal Instability (CIN) Pathway

Chromosomal instability refers to a significant increase in the gain or loss of either the entire or the large portions of chromosomes and is the most commonly occurring genetic instability in CRC. CIN is found in around 85% of adenocarcinoma transitions [16,17] and is characterized by the activation of oncogenes (*KRAS* and *BRAF*), inactivation of TSGs (*APC* and *TP53*), and a loss of heterozygosity for the long arm of chromosome 18 (18q LOH), thus, promoting CRC tumorigenesis [15,16].

According to the multistep genetic model defined by Fearon and Vogelstein [10], the first step includes the silencing of *APC*, followed by oncogenic *KRAS* mutations in the adenomatous stage, and, finally, deletion of chromosome 18q and inactivation of *TP53* occurring during the transition to malignancy (Figure 1) [10,18,19]. In addition, recently, genetic aberrations in *TGF-βR* and *PI3KCA* were found to be involved in the adenocarcinoma sequence model [20,21].

Furthermore, array-based comparative genomic hybridization and single nucleotide polymorphism techniques have identified a few frequently recurring allele losses of all chromosomal arms in CRC that include losses at chromosomal arms 1p, 5q, 8p, 17p, 18p, 18q, 20p, and 22q [3,22,23,24]. On the other hand, chromosomal gains were identified at chromosome 7 and chromosomal arms 1q, 8q, 12q, 13q, and 20q [3,22,23,24]. The allelic loss or gain of material denotes presence of candidate TSGs or oncogenes, respectively, thus allowing growth of mutated cells, leading to transformation of normal cells into cancerous [25].

#### 2.1.1. *Adenomatous Polyposis Coli* (*APC*) Gene and Wnt Signaling Pathway

The *APC* gene, located on chromosome 5q21-q22, consists of 8535 nucleotides spanning 21 exons and encodes a 310 kDa protein [26]. Around 75% of the coding sequence is present on exon 15, the most frequent region for both germline and somatic mutations of *APC* [26]. Furthermore, APC is a multi-domain protein. From the N to C-terminus, it consists of oligomerization, armadillo repeat, 15- or 20-residue repeat, SAMP repeats, a basic domain, and C-terminal domains [27]. Since *APC* interacts with various binding proteins through its different domains, *APC* plays a role in regulating cellular processes including chromosome segregation, cell migration, apoptosis, adhesion, proliferation, and differentiation [28,29,30,31,32].

*APC* mutations are present at the preliminary stages of neoplasia and are majorly linked with the classic tubular adenoma pathway and CIN cancers [33,34,35,36]. *APC* germline mutations give rise to familial adenomatous polyposis (FAP) syndrome (OMIM#: 608456), attenuated FAP, Gardner syndrome (OMIM#: 175100), Turcot syndrome (OMIM#: 276300), and other major hereditary predisposition events leading to CRC development [37]. FAP, an autosomal-dominant genetic disorder, is characterized by the development of around 1000 precancerous colon polyps in the large bowel in individuals aged 10–25 years. Germline mutation is found in 60–80% of families with FAP [38,39]. CIN is also known to stimulate tumorigenesis in FAP [40,41,42,43]. The attenuated form of FAP (AFAP) is marked by less than 100 adenomas or polyps in the more proximal part of the colon. Germline mutations occur in the 5′ or 3′ region of the *APC* gene [44]. More importantly, 16–40% of patients with less than 100 polyps present the biallelic inactivation of the MUTYH-based excision repair gene, a condition called MUTYH-associated polyposis (MAP) [45]. AFAP and MAP are phenotypically very alike [45]. While Gardner syndrome (OMIM#: 175100) is the subtype of FAP and is linked with osteomas and soft tissue tumors, Turcot syndrome (OMIM#: 276300) is characterized by colonic polyps along with tumors of the central nervous system [44]. On the other hand, somatic *APC* mutations (frameshift or nonsense mutations) are present in more than 70–80% of sporadic CRCs and 5q LOH is reported in approximately 40% of CRC cases [46,47,48].

In both familial and sporadic CRCs, the APC/β-catenin/Wnt-Tcf pathway plays a major role in the onset and progression of CRC carcinogenesis [49]. The *APC* gene inhibits transition from the G0/G1 to the S phase of the cell cycle. The Wnt signaling pathway maintains the undifferentiated stem cells in the base of the colonic crypts, allowing survival of both normal and cancer stem cells [32]. In this regard, it is well known that β-catenin is the main controller of the Wnt signaling pathway [50]. The wild-type APC protein negatively controls the Wnt signaling by regulating the ubiquitin-mediated proteasomal breakdown of the transcription factor β-catenin [51,52]. Disruption of the APC protein results in enhanced Wnt signaling by intracellular β-catenin stabilization, which stimulates transcription of Wnt targeted genes and enhances TCF targets with increase in cell growth, differentiation, spread, and adhesion of colorectal cells [53]. Mutations in genes involved in APC/β-catenin/Tcf pathway in CRC cells without *APC* mutations are also present in sporadic CIN tumors. Activating mutations in the gene for β-catenin (*CTNNB1*) block *APC*-regulated breakdown and are present in colorectal neoplasia. Although *CTNNB1* mutations are more common in adenomas (12.5%) than invasive cancer (1.4%), they are found in the preliminary stages of CRC pathogenesis and plausibly substitute *APC* mutations in cancer onset and progression [54,55,56]. In addition, distinct units of the APC/β-catenin/Wnt pathway can be either directly or indirectly changed, by constitutively triggering β-catenin or Tcf. Among the different regulatory genes that act with *APC*, the mitotic checkpoint protein BubR1 plays a vital role [28]. BubR1 is a part of the mitotic checkpoint machinery along with Bub1, Bub3, Mad1, Mad2, Mad3, Mps-1, CENP-E, and cell division cycle 20 (CDC20) [57]. Binding of BubR1 to Cdc20 blocks APC activity by triggering a “wait anaphase” signal [58,59], thus contributing to the development of polyploid cells, extended cell survival, and uncontrolled cell proliferation, suggesting a plausible pathogenic mechanism in the initiation of CIN in CRC sporadic forms [60].

Recently, activation of leucine-rich repeat-containing G-protein-coupled receptors (LGR-4 and LGR-5) triggered signaling by binding with proteins in the R-responding family [61,62,63,64]. Moreover, *cyclin D1* (*CCND1*) was implicated in APC signaling: Mutated APC cells activate downstream targets, such as *cyclin D1* and *Myc* [65,66]. *CCND1*, along with other cyclin-dependent kinases (CDKs) that block cyclins, such as *p27* (CDKN1B) and *p21* (CDKN1A), are vital for cell growth and apoptosis as well as cell cycle control, majorly during the transition from G1 to S phase [67]. Prolonged activation of *CCND1* by *APC* mutation leads to the onset of colonic neoplasia by allowing the cell to divert from apoptosis [68]. Arber and colleagues (1997) studied the expression of *CCND1* in normal colonic mucosa, adenoma, and adenocarcinoma and confirmed its high expression in colonic tumors, thus indicating *CCND1* expression as an early event during multistage process of CRC tumorigenesis that may deregulate cell-cycle control in benign adenomas and stimulate tumor progression [69].

On the other hand, β-catenin activity can be indirectly triggered by aberrations in oncogenes controlling its activity at different levels. β-catenin mutually interacts with various members of the Notch pathway that are vital regulators of cell differentiation and play a role in CRC carcinogenesis [70,71]. Kwon and colleagues showed that *Notch1* stimulates the assembly of active β-catenin protein in the absence of ligand-receptor activation [72]. Further genetic changes that modulate β-catenin activity include *CDK8* (*cyclin-dependent kinase-8*) gene amplification and occurs in over 60% of CRC cases. Increased CDK8 enhances both *β-catenin* [73] and *Notch1*, thus stimulating transcription and cell differentiation [73,74].

#### 2.1.2. TP53 Pathway

The *TP53* gene, located on the short arm of chromosome 17, is known as the “guardian of the genome” and encodes proteins regulating cell cycle, DNA repair, senescence, and apoptosis [75]. *TP53* mutations or loss of function are reported in 50–75% of CRC cases [76]; loss of *p53*-mediated pathways of apoptosis is an important determinant of progression from adenoma to malignant tumor [77]. The *p53* loss of function enhances high cell proliferation activities and uncontrolled cell cycle, a key step in colorectal carcinogenesis [77]. Research showed missense mutations (48%) that substitute AT for GC as the most common *TP53* mutations in CRC [78,79], followed by point mutations (37.5%) with transitions at CpG sites [79].

Commonly, *p53* is considered a controller for BubR1 transcription and expression; loss of *p53* expression downregulates BubR1, thus comprising checkpoint function to mitotic aberrations leading to progression of CRC [80]. Additionally, a wild type of p53 is identified as a direct activator for *WAD-1*, a gene highly induced to suppress tumor cell growth in the p53 pathway [81].

Moreover, mutations in *CDK* are likely to occur during CRC development and progression [82]. Adenosine monophosphate-activated protein kinase (AMPK) pathway induces phosphorylation of *p53* and induces AMPK-dependent cell-cycle arrest that upregulates *the cyclin-dependent kinases inhibitor 1A* (*CDKN1A* or *p21*). This eventually controls cell cycle regulation, cellular senescence, and stem cell aging [83]. Furthermore, in CRC, the stimulation of *p21* occurs in a p53-dependent pathway; *p21* also inhibits the activity of cyclin D1 [84]. Loss of *p21* is significantly associated with poor prognosis in CRC [84]. Another type of CDK associated with *TP53* loss of function is *CDK inhibitor 1B* (*CDKN1B* or *p27*) [85]. Specifically, p27 is an enzyme inhibitor that encodes CDK inhibitor proteins responsible for regulating cell cycle progression into S phase and its degradation [85]. It has been established that p27 expression is inversely related with the MSI-H and CIMP-H types of CRC and *TP53*-negative cancers [86].

The p53 also interacts with *cyclooxygenase-2* (*COX-2*) and is involved in enhancing inflammation and CRC cell proliferation [87]. Interestingly, *COX-2*-positive tumors are significantly linked with cancer-specific mortality regardless of p53 status, thus suggesting *COX-2* as an independent CRC prognostic factor [88,89].

#### 2.1.3. The 18q Loss of Heterozygosity (LOH)

Loss of heterozygosity (LOH) refers to the absence of one of the two copies or alleles of a gene, with the remaining allele frequently being affected by mutation [44]. LOH in the 18q region is most commonly observed in advanced CRC, accounting for approximately 70% of the cases [90], and is associated with poor prognosis in CRC [91,92]. LOH at 18q indicates presence of several TSGs including *Deleted in Colorectal Carcinoma* (*DCC*), *SMAD2,* and *SMAD4*; loss of expression of 18q LOH plays a significant role in CRC pathogenesis [19].

*DCC*, located on chromosome 18q21.2, encodes netrin-1 and is indicated as a plausible TSG [93]; LOH in the *DCC* gene region is present in approximately 70% of CRCs. Moreover, a few somatic mutations in *DCC* are found in CRC [94]. Netrin-1 is built within the cryptos of colorectal mucosa; epithelial cell differentiation results in loss of netrin-1 expression [95]. Furthermore, mutation in *DCC* gene inhibits binding of netrin-1 to DCC transmembrane protein, leading to abnormal cell survival [95].

On the other hand, *SMAD2* and *SMAD4* are present on 18q21.1, the prevalent region lost during CRC progression, and correlate with adenoma development and adenocarcinoma progression in mice models, thus suggesting a plausible tumor suppressor role for *SMAD* genes [19,96]. Furthermore, immunohistochemical analysis reported a loss of SMAD4 expression in >50% of CRCs, which is associated with lymph node metastases [97]. Since the frequency of somatic mutations in *SMAD2* and *SMAD4* is comparatively low in CRC [98], other TSGs might be responsible for chromosome 18q loss. Research has indicated that *SMAD* genes encode for TGF-β [99]. Dysregulation of TGF-β signaling occurs in the majority of CRCs [36]. Additionally, inactivating mutations in receptor genes including *TGF-βR1, TGF-βR2,* and TGF-β superfamily members Activin Receptor type 2 (ACVR2) are reported in CRC [100]. Functionally, marked mutations in *TGF-βR2* are present in ~30% of all CRC cases and correlate with malignant transformation of late adenomas. *TGF-βR2* mutations are most frequent in MSI tumors; however, they are also present in around 15% of MSS tumors [12,20,101].

### 2.2. The Microsatellite Instability Pathway

Another type of genomic instability in CRC is microsatellite instability (MSI), a distinctive characteristic of cancerous cells [102]. MSI is the hallmark of HNPCC or Lynch syndrome and occurs in >95% of HNPCC cases [103]. However, in the majority of sporadic CRCs, the underlying mechanism for CIN remains nascent and MSI comprises merely 15–20% of all CRC cases [103].

A subset of tumors with unstable loci in ≥30% markers are defined as “Microsatellite high” (MSI-H), a subset of tumors with 10–29% unstable loci are classified “Microsatellite low” (MSI-L), and “microsatellite stable” (MSS) is marked with no unstable markers [104]. In cancers with MSI-H, small insertions/deletions result in frameshift mutations within repetitive tracts in the coding regions of TSGs or oncogenes, further contributing to tumorigenesis [105]. Mori et al. [106] performed large-scale genomic screening of the coding region of microsatellites and found mutations in nine loci (*TGF-βR2, Bax, MSH3, ActRIIB, SEC63, AIM2, NADH-ubiquinone oxidoreductase, COBLL1,* and *EBP1*) in >20% of tumors. TGF-βR2 was the most commonly mutated loci and instability in the poly-adenine tract of TGF-βR2 is present in approximately 85% of MSI-H CRCs [107]. Moreover, Bax, the other frequently mutated gene, was found to have frameshift mutations within the poly-guanine sequence in almost 50% of the MSI-H CRCs, resulting in the inactivation of Bax and inhibition of apoptosis [108].

MSI is rarely found in polyps, except in Lynch syndrome [109,110]. Furthermore, individuals with Lynch syndrome frequently develop MSI CRCs due to germline mutations in one of the MMR genes (*MLH1, MSH2, MSH6,* and *PMS2*); mutations in *MLH1* or *MSH2* gene lead to an increased risk (70–80%) of developing cancer, while mutations in the *MSH6* or *PMS2* gene have a comparatively lower risk (25–60%) of cancer development [111] On the contrary, sporadic MSI CRCs frequently display loss of MMR activity due to *MLH1* silencing by aberrant DNA methylation [112,113].

Furthermore, according to modeling studies and absence of colon cancer in individuals with biallelic germline mutations in MMR genes suggest that lack of MMR activity is insufficient to trigger polyp formation [114,115]. Relevant to its clinical impact, there is considerable indirect data that polyps arising as a result of MMR activity loss has a lesser transition interval from a polyp to colorectal cancer; polyps can develop into MSI CRCs in as less than 2–3 years [116]. Evidence suggesting lack of MMR progress and onset of MSI induces tumor development and progression is based on the fact that MSI is generally observed in polyps adjacent to cancers and is infrequent in non-advanced polyps [116]. It is known that sporadic MSI CRCs correlate with the serrated neoplasia pathway and commonly carry *BRAFV600E* mutations, on the contrary, Lynch syndrome arises from MMR genes germline mutations and lacks mutated *BRAF* [117,118]. 

Clinically, *BRAF*-mutated CRC correlates with poor prognosis and overall survival (OS) in comparison to *BRAF* wild-type disease [119]. Furthermore, a study showed that *BRAF-*mutated CRC patients had worst OS as compared to patients carrying *RAS* (*KRAS* and *NRAS*) mutations [120]. Also, mutation in *BRAF* is a negative prognostic factor in stage II and III disease [121]. On the contrary, a recent study, performed meta-analysis in 1164 MSI-H non-metastatic CRC patients and showed that *BRAF* V600E mutation is associated with worst OS, but not disease recurrence [122]. Moreover, another meta-analysis in patients undergoing resection of liver metastasis showed that following metastasectomy, OS was worst for *BRAF-*mutated metastatic CRC [123]. It has also been revealed that, non-V600E *BRAF-*mutated (*BRAF* codons 594 and 596) CRCs have better prognosis as compared to *BRAF-*mutated CRCs; *BRAF* 594 and 496 tumors are microsatellite stable, rectal, non-mucinous with no peritoneal spread and have a significantly longer OS as compared to V600E *BRAF-*mutated tumors [124]. Similarly, other studies in CRC patients showed, in comparison to V600E *BRAF-*mutated tumors, non-V600E *BRAF-*mutated tumors are present in the younger population, lower grade tumors and the median OS is significantly longer compared with both V600E *BRAF*-mutant and *BRAF* wild-type patients [125,126].

### 2.3. CpG Island Methylator Phenotype (CIMP) Pathway

DNA methylation is the addition of a methyl group to cytosine in the 5′-position that is catalyzed by DNA methyltransferases via covalent linkage within a CG dinucleotide sequence within the promoter region, termed CpG transcription [127,128]. In normal cells, the majority of the CpG sites are heavily methylated while CpG islands, usually located in the promoter regions of genes, are unmethylated. However, following cancer initiation, hypermethylation within the promoter region may lead to inactivation of tumor-suppressor genes, while global hypomethylation is associated with genomic instability and chromosomal aberrations [129]. Epigenetic instability in CRC is demonstrated as hypermethylation of loci that contain CpG islands and this is usually accompanied by global DNA hypomethylation. Alterations in methylation pattern can affect virtually all signaling pathways, including TP53, TGFβ/SMAD, Wnt, NOTCH and receptor tyrosine kinases involved in cell cycle regulation, transcription regulation, DNA stability, apoptosis, cell-cell adhesion, angiogenesis, cell invasion and metastasis [130,131,132].

Many genes are identified to be methylated and silenced in CRC, some commonly methylated ones include *APC, MLH1, MGMT, SFRP1, SFRP2, CDKN2A, TIMP3, VIM, SEPT, CDH1* and *HLTF*. Additionally, there is a distinct subset of CRCs, known as the CpG island methylator phenotype (CIMP) [133]; CIMP tumors frequently carry *BRAFV600E* mutations [134]. CIMP is further subclassified based on integrated genetic and epigenetic instability into CIMP2, CIMP-low, and CIMP-high [135,136]. DNA methylation profiling revealed that approximately 20% of CRCs are CIMP tumors; CIMP tumors significantly correlated with age, female sex, proximal colon location, as well as *MSI, KRAS* and *BRAF* mutations [87,137]. The most commonly used CIMP markers are *MLH1, p16, MINT1, MINT2* and *MINT31*. Additional markers include *CACNA1G, CRABP1, IGF2, NEUROG1, RUNX3, SOCS1, HIC1* and *IGFBP3* for positive CIMP identification [138,139].

Although, upregulated expression of the DNA methyltransferases (DNMT3B or DNMT1) is associated with CIMP, the underlying mechanism(s) that promote CIMP are still unknown [140]. One of the plausible underlying mechanisms is based on the silencing of barriers that inhibit methylation of normally unmethylated CpG islands [141,142]. The other suggested mechanism is alterations in the chromatin structure and histone modification state of histone H3 lead to the detection of aberrant DNA methylation in loci that obtain this alteration [143,144]. While, *PTEN*, a TSG, shows reduced methylation rates, *TWIST1* gene is silenced by promoter methylation in CRC. Aside from all of the above-mentioned mechanisms of DNA hypermethylation, global DNA hypomethylation frequently occurs at repetitive sequences including LINE-1 repeats, retrotransposons, introns and gene deserts [133,145].

#### EGFR-KRAS-BRAF Pathway

The epidermal growth factor receptor (EGFR) belongs to the ErbB/HER family and consists of four members; ErbB1 (EGFR/HER1), ErbB2 (Neu/HER2), ErbB3 (HER3), and ErbB4 (HER4) [146,147]. Activation of EGFR pathway, triggers several downstream intracellular signaling pathways, including the RAS/RAF/MEK/ERK, PI3K/AKT, and JAK/STAT3 pathways to regulate cell growth, survival, and migration [148,149,150]. Deregulated *EGFR* expression is present in various cancers including CRC [146]; increased *EGFR* expression is present in 25–77% of CRC cancers [151,152]. Activation of EGFR induces RAS-RAF activation, which leads to phosphorylation of mitogen-activated protein kinase (MAPK or MEK) and activation of extracellular signal-related kinase (ERK) [153,154]. 

The MAPK pathway includes *KRAS* and *BRAF*; the RAS/RAF/MAPK pathway is involved in regulating cell proliferation, differentiation, apoptosis and senescence [153]. Activation of MAPK includes RAS, RAF and MEK; RAS stimulates the signaling cascade via the phosphoinositol kinases (PI3K) as well as RAF [155,156]. PI3K activation inhibits apoptosis, activation of *RAS* provokes cellular proliferation, thus, promoting cell survival and tumor invasion and metastasis [156,157]. *KRAS* mutations include codons 12 and 13 on exon 2 and codon 61 on exon 3; codon 12 being the most frequently affected through missense mutations [158] including substitution of glycine for aspartate (p.G12D and p.G13D) [159], of which p.G13D account for 58% of the cases [160]. *KRAS* along with *NRAS* and *HRAS* are oncogenes belonging to the RAS family [161]. *KRAS* is commonly mutated in sporadic CRCs (35–45%) [158,162,163] and is associated with poor prognosis [164,165,166]; according to the adenocarcinoma sequence, *KRAS* mutations occur after APC mutations [10]. 

On the other hand, *BRAF*, a member of RAF family of serine/threonine kinases regulates cellular responses to growth signals through the RAS-RAF-MAP kinase pathway [167]. Activating mutations in *BRAF* are found in approximately 5–10% of metastatic CRC, however, they are rare in Lynch syndrome forms of CRC [167,168]. Moreover, *BRAF* mutations were identified in 40% of MSI-H and 4% of MSI-L tumors [169]. The majority of the *BRAF* mutations include the hotspot mutation, V600E (Val600Glu) and is found to correlate with poor prognosis in CRC patients [170,171].

**Figure 2:**
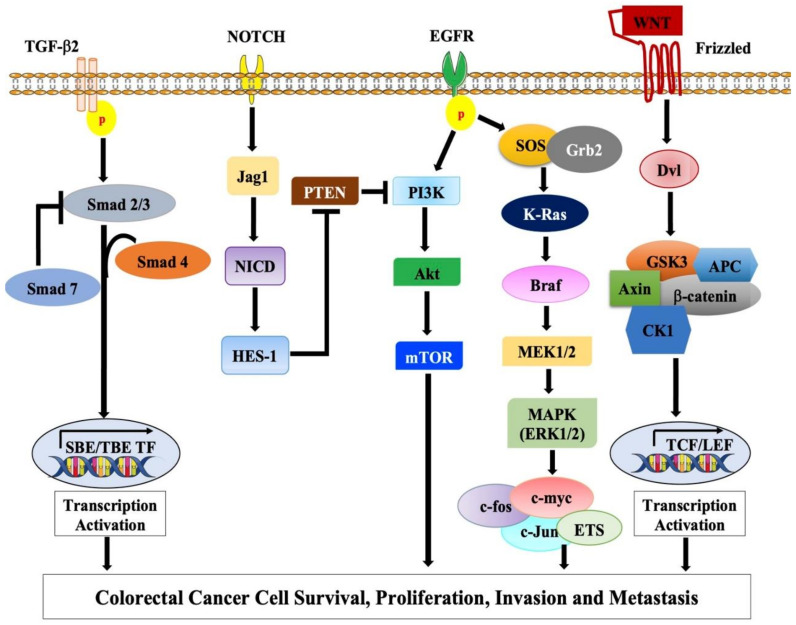
Schematic representation of the molecular pathways involved in CRC pathogenesis.

Angiogenesis, the development of new blood vessels, is involved in tumor initiation, growth, and metastasis and involves several factors including vascular endothelial growth factors (VEGFs). In CRC, VEGF levels and VEGFR activity is enhanced and is associated with poor prognosis [172]. Elevated VEGF levels are seen in very early stages of colorectal neoplasia (adenoma); however, they were significantly elevated in a later stage of cancer (metastatic stage) [173]. Aberrant *KRAS* and *TP53* as well as *COX-2* expression regulate VEGF-VEGFR activity alteration, thus promoting cancer growth and migration [173,174]. The molecular pathways involved in the pathogenesis of CRC are depicted in Figure 2.

## 3. Therapeutic Strategies

Molecular profiling has helped in developing biomarkers that can potentially improve clinical outcomes in CRC patients and significantly enhance the survival of metastatic patients. Several molecular aberrations are defined for candidate biomarkers that have been tested in completed and ongoing trials in conjunction with targeted therapies and immunotherapies.

### 3.1. Targeting CIN Pathway

There are intense efforts to develop synthetic modulators of Wnt signaling including small molecules, peptides, and blocking antibodies to inhibit Wnt pathway [175]. As approved by US Food and Drug Administration (FDA), lithium chloride is already in clinical use and it is found to stimulate *CTNNB1* by inhibiting GSK3. Moreover, non-steroidal anti-inflammatory drugs (NSAIDs) and celecoxib, the selective *COX-2,* block *CTNNB1*-dependent transcription in CRC [176,177] and lessen polyp formation in FAP patients as well in in vivo mice models of colon cancer [178,179]. Recently, two small molecules, XAV939 and pyrvinium, were identified using reporter-based screening approaches, while XAV939 increased AXIN stability by inhibiting tankyrase, pyrvinium stimulated CTNNB1 phosphorylation through casein kinase activation [180,181]. On the other hand, several Wnt-blocking antibodies blocked proliferation and induce apoptosis in different cancers [182]. In comparison to normal tissues, FZD7-specific antibodies selectively target colon cancer and HCC cells [183,184]. In vivo studies have shown that intraperitoneal injections of WNT3A-neutralizing antibodies reduce proliferation and increase apoptosis of prostate cancer in mouse models [185]. In addition, studies have indicated that use of Wnt-modulatory peptides, SFRP1, or SFRP1-derived peptides reduce HCT116 xenograft tumor development in nude mice [186,187].

On the other hand, no definite clinical role for TGF-β signaling pathway has been identified thus far; however, studies show that *SMAD4* expression levels correlate with prognosis and response to 5-fluorouracil (5-FU) [188,189,190]. Moreover, current clinical trials (e.g., NCT00217737, also designated ECOG 5202) are studying the benefit of 18qLOH for directing the use of specific adjuvant therapies [72]. They also found that chronic use of NSAIDs, mainly Ibuprofen, stimulates a dose-dependent reduction of Notch pathway activity, thus confirming the protective effects of NSAIDs in CRC [72]. Remarkably, a few clinical trials aimed at targets including IGF-1R, Wnt, Notch, Hedgehog, and TGF-β are in progress; however, no definite results have appeared thus far. Phase II trials of the γ-secretase inhibitor (RO4929097) in Notch blockade therapy and the Hedgehog pathway inhibitor vismodegib can be considered promising [191,192].

### 3.2. Targeting MSI Pathway

MSI status has been demonstrated to be a reliable biomarker of immunotherapy response in the metastatic setting. Currently, checkpoint inhibitors are investigated in various solid tumors with promising responses [193,194]. Pembrolizumab, a humanized IgG4 antibody, was the first PD-1 blocker approved by FDA for treatment of metastatic CRC [193]. Another phase I trial in patients with MSI-H CRC found antitumor activity of pembrolizumab [194]. Although combination therapy of pembrolizumab and ipilimumab showed comparable efficacy in melanoma patients, the effect of this combination in CRC is still nascent [195]. The other humanized monoclonal IgG4-based PD-1 antibody, nivolumab, gained FDA approval for MSI-H metastatic CRC based on the results obtained in the CheckMate-142 clinical trial [196]. Remarkably, a combined therapy of nivolumab and ipilimumab improved the outcome of the patients with dMMR or MSI-H CRC who had previously received chemotherapy [197]. Other plausible candidate PD-1/PD-L1 inhibitors (atezolizumab, avelumab, and durvalumab) are under phase I trials for several tumors including CRC [198]. Furthermore, new immune checkpoint targets including TIM-3, TIGIT, T cell Ig, and T cell-derived LAG-3, which promote CRC progression, are also in phase I trials [199,200].

### 3.3. Targeting CIMP Pathway

Targeting the EGFR pathway generally involves use of anti-EGFR monoclonal antibodies and tyrosine kinase inhibitors (TKIs) aimed at intracellular kinases. Cetuximab was the first monoclonal antibody introduced to target EGFR. Cetuximab provokes EGFR internalization and degradation once bound to the external domain of EGFR [201]. Multiple studies have confirmed the beneficial effects of the cetuximab on CRC patients’ outcome [202]; in addition, a combined therapy of cetuximab with other existing chemotherapies also showed favorable results [203]. However, tumors carrying *RAS*, *BRAF,* or *PIK3CA* mutations, loss of *PTEN*, *HER-2* amplification, and altered VEGF and VEGFR signaling are resistant to anti-EGFR therapy due to continuous activation of EGFR downstream signaling pathways [204]. In these cases, the patient is not eligible for anti-EGFR treatment with cetuximab. The other EGFR target, panitumumab, is a fully humanized antibody and, in comparison to cetuximab, does not provoke antibody-dependent cell-mediated cytotoxicity [205]. In the PRIME trial, efficacy of FOLFOX (folinic acid, fluorouracil, and oxaliplatin) alone as well as in combination with panitumumab was analyzed in patients with metastatic CRC. The combined therapy showed a higher OS and PFS than for FOLFOX alone [206,207]. PRIME and PEAK trials further analyzed the effects of panitumumab and fluorouracil/leucovorin (5-FU/LV) after panitumumab plus FOLFOX. The trials showed significant improvement in PFS and OS compared with panitumumab treatment alone [208]. In addition, the VALENTINO trial demonstrated synergistic effects of panitumumab with 5-FU/LV and better effects on outcome compared with treatment with panitumumab alone [209]. Although both cetuximab and panitumumab are FDA-approved drugs, panitumumab is economically more effective than cetuximab [210].

On the other hand, there are no approved specific targeted therapies for *KRAS*-mutated CRC. However, a novel agent, AMG 510, which is a small molecule that targets KRASG12 C mutation, was introduced. AMG 510 specifically and irreversibly blocks KRASG12 C by locking it in the inactive GDP-bound state [211]. In addition, other *KRAS*-modulating agents targeting G12C (MRTX849, LY3499446, or ARS-1620) and G12D mutations are also under investigations [212,213].

Although *BRAF* mutations are more frequent in melanoma and papillary thyroid carcinoma in comparison with CRC, a few studies analyzed the effects of *BRAF* inhibitors (vemurafenib or dabrafenib and trametinib) in patients with metastatic CRC. Although downstream MAPK activity was inhibited, the PFS or OS of patients did not improve [214,215,216]. However, the synergistic effect of *BRAF* and *EGFR* inhibitors in trials using vemurafenib in combination with IRI and cetuximab in *BRAF*-mutant CRC patients showed positive results [217,218,219,220]. Additionally, in a phase II trial, using encorafenib (a BRAF inhibitor) along with cetuximab, with or without alpelisib (ALP), improved the OS and PFS in advanced *BRAF*-mutant CRC patients [221]. Furthermore, a study by Corcoran et al. [218] found that patients with *BRA*F-V600E-mutated CRC, when treated with a triplet regimen (dabrafenib, trametinib, and panitumumab), produced a better response rate in comparison with the double regimens (dabrafenib + panitumumab or trametinib + panitumumab). The ongoing BEACON trial, which employs the triplet regimen of encorafenib, binimetinib (a MEK inhibitor), and cetuximab, has shown less toxicity and higher survival rates [222].

Anti-VEGF/VEGFR therapies are necessary to target steps in tumor metastasis. Bevacizumab (Avistin), a humanized IgG monoclonal antibody, was the first FDA-approved VEGF-targeted agent for metastatic CRC. According to several trials, bevacizumab showed to improve both progression-free survival and OS in metastatic CRC [223,224]. Furthermore, *KRAS-*mutant patients were also found to benefit from bevacizumab [225,226]. Moreover, combination of FOLFOX and bevacizumab improved progression-free survival and OS in CRC patients as compared to treatment with FOLFOX alone [227].

## 4. Conclusions

This brief review provides information about candidate biomarkers that can aid in improving the diagnosis and help with the early detection of CRC cases, thus ameliorating the prognosis of CRC patients. Although there are marked advances in CRC investigations, the role of molecular classification in therapeutic interventions remains unclear. The use of molecular alterations in predicting risk for CRC shows promise, while further studies are needed to determine if aberrantly methylated CpGs or other molecular alterations can be used as reliable and accurate indicators of risk for polyps or CRCs. Moreover, it is important to analyze the efficacy of multi-kinase/BRAF-inhibitor, sorafenib, in addition to other specific inhibitors of the EGFR as well as PI3K signaling pathway, in the treatment of CRC to further identify novel therapeutic targets. On the other hand, we believe that using new drug combinations and specific (personalized) targets will be the future avenue for efficient treatment of CRC patients with limited risk of toxicity and adverse side effects. Thus, understanding the underlying mechanisms of CRC cell genetic alteration and subsequent consequences can help pave the way for the development of novel diagnostic and therapeutic strategies.

## Figures and Tables

**Table 1:** Biological differences in the gene expression-based molecular subtypes of colorectal cancer (CRC).

Subtype	Gene Expression	Prognosis	Reference
CMS1 (MSI immune)	Deregulated DNA mismatch repair, MSI and MLH1 silencing CIMP-high with common *Serine/threonine-protein kinase B-Raf* (*BRAF*) mutations and low number of SCNAs Immune infiltration and activation	Very poor survival rate after relapse	[4]
CMS2 (Canonical)	Express epithelial signatures with Wingless (Wnt) and *MYC* signaling activation Frequently exhibit loss of TSGs and overexpression of oncogenes than the other subtypes	Better survival rate after relapse in comparison to other subtypes	
CMS3 (Metabolic)	Fewer SCNAs as compared to CMS2 and CMS4 Epithelial signatures Metabolic dysregulation in a variety of pathways with frequent *KRAS* mutations Slightly higher presence of CIMP-low	Better survival rate after relapse in comparison to other subtypes	
CMS4 (Mesenchymal)	Activation of Transforming growth factor-β (TGF-β) Upregulated expression of EMT genes Enhanced expression of genes regulating inflammation, matrix remodeling, stromal invasion and angiogenesis	Worse overall and relapse-free survival as compared to other subtypes	

CIMP: CpG island methylator phenotype; EMT: epithelial-mesenchymal transition; MSI: microsatellite instability; SCNA: somatic copy number alterations.

## Abbreviations

5-FU	Fluorouracil
ACVR2	Activin receptor type 2
AFAP	Attenuated familial adenomatous polyposis
ALP	Alpelisib
AMPK	Adenosine monophosphate-activated protein kinase
APC	Adenomatous polyposis coli
BRAF	Serine/threonine-protein kinase B-Raf
Bub	Budding uninhibited by benzimidazoles
CDC20	Cell division cycle 20
CDKN	Cyclin-dependent kinases
CIMP	CpG island methylator phenotype
CIN	Chromosomal instability
CMS	Consensus molecular subtypes
COX	Cyclooxygenase
CRC	Colorectal cancer
CTNNB1	Catenin beta-1
DCC	Deleted in colorectal cancer
dMMR	Defective mismatch repair
EDM	Exonuclease domain
EGFR	Epidermal growth factor receptor
ERK	Extracellular signal related kinase
FAP	Familial adenomatous polyposis
FDA	Food and Drug Administration
FOLFOX	Folinic acid, fluorouracil, and oxaliplatin
GSH	Glutathione
GSK3-β	Glycogen synthase kinase 3-β
HNPCC	Hereditary non-polyposis colorectal cancer
IBD	Inflammatory bowel disease; Ig: Immunoglobin
IGFBP7	Insulin-like growth factor-binding protein 7
JAK	Janus kinase
KRAS	Kirsten ras
LOH	Loss of heterozygosity
LV	Leucovorin
MAP	MUTYH-associated polyposis
MAP/MEK	Mitogen-activated protein kinase
MLH/MSH	MutL homolog
MSI	Microsatellite instability
MSS	Microsatellite stable
NF-κB	Nuclear factor kappa
NSAIDs	Non-steroidal anti-inflammatory drugs
OS	Overall survival
PD-1/PD-L1	Programmed death ligand-1
PI3K	Phosphatidylinositol 3-kinase
PI3KCA	Phosphatidylinositol-4,5-Bisphosphate 3-Kinase Catalytic Subunit Alpha
POLE/POLD1	Polymerase epsilon or delta 1
RAC1	Rac family small GTPase 1
ROS	Reactive oxygen species
SCNAs	Somatic copy number alterations
SMAD	Small mothers against decapentaplegic
STAT	Signal transducer and activator of transcription
TCGA	The Cancer Genome Atlas
TGF-β	Transforming growth factor-β
TIGAR	T53-induced glycolysis regulatory phosphatase
TNM	Tumor-nodes-metastasis
TP53	Tumor protein 53
TSG	Tumor suppressor gene
VEGF	Vascular endothelial growth factor
VEGFR	Vascular endothelial growth factor receptor
Wnt	Wingless
